# GCK-MODY (MODY 2) Caused by a Novel p.Phe330Ser Mutation

**DOI:** 10.5402/2011/676549

**Published:** 2011-04-26

**Authors:** Walter Bonfig, Sandra Hermanns, Katharina Warncke, Gabriele Eder, Ilse Engelsberger, Stefan Burdach, Annette Gabriele Ziegler, Peter Lohse

**Affiliations:** ^1^Division of Pediatric Endocrinology, Department of Pediatrics, Technische Universität München Kölner Platz 1, 80804 Munich, Germany; ^2^Institut für Diabetesforschung, Helmholtz Zentrum München, 85764 Neuherberg, Germany; ^3^Department of Clinical Chemistry, Klinikum Großhadern, Ludwig Maximilian University of Munich, 81377 Munich, Germany

## Abstract

Maturity onset diabetes of the young (MODY) is a monogenic form of diabetes inherited as an autosomal dominant trait. The second most common cause is GCK-MODY due to heterozygous mutations in the *GCK* gene which impair the glucokinase function through different mechanisms such as enzymatic activity, protein stability, and increased interaction with its receptor. The enzyme normally acts as a glucose sensor in the pancreatic beta cell and regulates insulin secretion. We report here a three-generation nonobese family diagnosed with diabetes. All affected family members presented with mild hyperglycemia and mostly slightly elevated hemoglobin A1c values. Genetic testing revealed a novel heterozygous T → C exchange in exon 8 of the *GCK* gene which resulted in a phenylalanine_330_ TTC → serine (TCC)/p.Phe330Ser/F330S substitution.

## 1. Introduction

MODY is a monogenic disease which accounts for 2–5% of all diabetes cases. The most frequent form is HNF-1*α*-MODY (MODY type 3), which is caused by mutations in the *HNF1A* gene encoding hepatic nuclear factor 1*α*. The second most frequent form is GCK-MODY (MODY type 2), which has been shown to be the result of mutations in the *GCK* gene [1]. The *GCK* gene maps to chromosome 7p15.3-p15.1 and consists of 12 exons that encode the 465-amino-acid protein glucokinase [2, 3], which is one of four members of the hexokinase family of enzymes. It catalyzes the phosphorylation of glucose as the first step of glycolysis. Glucokinase is exclusively expressed in mammalian liver and pancreatic islet beta cells. The enzyme plays an important regulatory role in glucose metabolism. As a glucose sensor, it regulates insulin secretion in the pancreatic *β*-cell by changing the glucose phosphorylation rate over a range of physiological glucose concentrations (4–15 mmoL/L; [4]). *GCK* gene mutations can cause both hypo- and hyperglycemia. Heterozygous inactivating mutations cause GCK-MODY, which mostly presents with mild hyperglycemia and is inherited in an autosomal dominant fashion [5, 6]. Usually, no diabetes-related complications such as nephropathy or retinopathy occur in patients with GCK-MODY. Homozygous or compound heterozygous inactivating *GCK* mutations result in a more severe phenotype presenting at birth as permanent neonatal diabetes mellitus [7–11]. Heterozygous activating *GCK* mutations, in contrast, cause persistent hyperinsulinemic hypoglycemia of infancy [12–16].

In 1992, *GCK* was the first MODY gene to be linked to disease in French and UK families [5, 6]. These linkage studies were quickly followed by the identification of the first genetic defects. Meanwhile, over 600 different *GCK* mutations have been described in many populations, the majority having been identified in Europe [17]. Missense, nonsense, frameshift, and splice site mutations have been reported, which are distributed throughout the 10 exons encoding the pancreatic *β*-cell isoform of the enzyme. There are no mutational hot spots. Over 250 mutations have been reported in more than one family [17]. All inactivating mutations are associated with mild fasting hyperglycemia while other specific symptoms are missing. Therefore, GCK-MODY is likely to be underdiagnosed, and no reliable data on its prevalence are available. In Caucasians, approximately 2% of the population are diagnosed as having gestational diabetes and approximately 2–5% of these patients will have GCK-MODY [18]. This would suggest a population prevalence of 0.04–0.1%.

The identification of a *GCK* gene mutation is important for the correct and definite diagnosis of GCK-MODY. It also helps the clinician to predict the clinical course of the disease and to advise appropriate therapy. Since only mild fasting hyperglycemia and no diabetes-related complications are usually present, diet is sufficient as a therapeutic approach in most cases [19].

We report a novel heterozygous inactivating *GCK* gene mutation in a nonobese family diagnosed with diabetes over three generations. 

## 2. Case Report

A 17-year-old, nonobese (BMI 24.3 kg/m²) female was admitted for the evaluation of recurrent syncopes. A blood glucose profile was obtained, and she was found to be mildly hyperglycemic (blood glucose values between 6.7 and 10 mmoL/L). Hemoglobin A1c was slightly elevated with 6.5% (normal range 4.0–6.0%) or 48 mmoL/moL (normal range 20–42 mmoL/moL). A standard oral glucose tolerance test with 75 g of glucose equivalent was performed (Table 1) with a fasting glucose of 6.5 mmoL/L and a 2-hour glucose of 14.6 mmoL/L. The fasting insulin concentration was 3.6 *μ*U/mL and 66.1 *μ*U/mL after two hours.

Her family history was strongly positive for diabetes. The patient's brother, her father, and the grandparents on her father's side were diagnosed with diabetes. Her grandmother was treated with oral antidiabetic medication (glimepiride). The index patient also reported that she had been diagnosed with mild hyperglycemia ten years ago, but this had no therapeutic consequences, and no followup analyses were performed.

This clinical presentation was highly suggestive of GCK-MODY. Therefore, a mutation analysis of the *GCK* gene was initiated. Genomic DNA of the patient was isolated, and PCR amplification of the pancreas-specific exon 1a as well as of exons 2–10 of the *GCK* gene was performed. Sequencing of the PCR products identified a novel phenylalanine_330_ (TTC) → serine (TCC)-/p.Phe330Ser-/F330S substitution encoded by exon 8. The index patient was also screened for type 1 diabetes-specific antibodies in the context of a Bavarian diabetes registry (DiMelli). Insulin autoantibodies (IAA), tyrosinephosphatase antibodies (IA2A), and zinc transporter antibodies (ZnT8c) were negative. Antibodies against glutamatedecarboxylase (GADA) were weakly positive.

**Figure 1:**
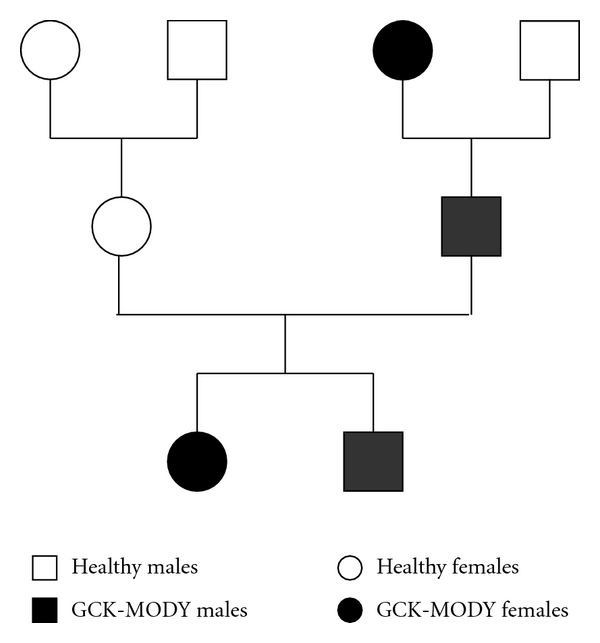
Circle: females, boxes: males, white: healthy, black: GCK-MODY.

Carrier screening of clinically affected family members revealed the same mutation in the pedigree with the exception of the grandfather, who has type 2 diabetes mellitus (Figure 1). The phenotype of the index patient's brother was comparable to that of the index patient (BMI 21.5 kg/m², hemoglobin A1c 6.2%, or 44 mmoL/moL). The father of the index patient was diagnosed with diabetes at 25 years of age. He also presented with very mild hyperglycemia and haemoglobin A1c at the upper end of the normal range (5.9% or 41 mmoL/moL). The father's BMI was 27.8 kg/m². Despite normal haemoglobin A1c, an antidiabetic treatment with metformin was started by the father's diabetologist. The index patient's grandmother was diagnosed with diabetes at the age of 62 years. Her BMI was 30.0 kg/m², and she had a haemoglobin A1c value of 7.0% or 53 mmoL/moL. She was treated with glimepiride. The grandfather was also diagnosed with diabetes but was not on any antidiabetic medication. His BMI was 29.8 kg/m² and his haemoglobin A1c never exceeded 7.0% or 53 mmoL/moL. No *GCK* mutation was found in him, so his final diagnosis is type 2 diabetes mellitus.

Because of the typical clinical symptoms and the cosegregation of the phenotype with the genotype, we conclude that this novel mutation is a pathogenic one and not a polymorphism without an effect on protein function. 

## 3. Discussion

So far, more than 620 *GCK* gene mutations have been reported in over 1400 patients with GCK-MODY (Table 2), permanent neonatal diabetes, and hyperinsulinemic hypoglycemia [17, 20, 21]. The mutations are spread over the ten exons of the gene which encode the pancreatic beta cell isoform of glucokinase (exons 1a and 2–10). Only few mutations have been detected in exon 1a, since most laboratories only screen for *GCK* mutations in exons 2–10 [18]. Missense, nonsense, frameshift, and splice site mutations have been reported. There are no mutation hot spots. Most of the mutations are private, although 255 mutations have been described in more than one family [18, 22]. We here report a novel heterozygous p.Phe330Ser mutation encoded by *GCK* exon 8 which results in the typical GCK-MODY phenotype. The mutation was found in three generations of a nonconsanguineous family, following an autosomal dominant inheritance pattern.

A *GCK* gene polymorphism with functional significance also has been reported in the literature [23, 24]. The G>A exchange at position −30 of the beta-cell-specific promoter (rs1799884) has been shown to be associated with increased fasting glucose levels in young adults and in pregnant women as well as an increased birth weight of their offspring [23].

The identification of a pathogenic *GCK* gene mutation is important for the correct and definite diagnosis of GCK-MODY and helps the clinician to predict the disease course and to initiate the appropriate therapy. Since only mild fasting hyperglycemia and usually no diabetes-related complications are present in patients with GCK-MODY, diet is sufficient as a therapeutic approach in most cases [19]. This may significantly improve the patient's quality of life. 

## Figures and Tables

**Table 1:** Glucose and insulin concentrations during a standard oral glucose tolerance test with 75 g glucose equivalent.

Time [min.]	Glucose [mg/dl]	Glucose [mmol/L]	Insulin [*μ*U/mL]
0	117	6.5	3.6
30	191	10.6	31.1
60	247	13.7	53.2
120	263	14.6	66.1

**Table 2:** Published GCK mutations causing GCK-MODY (MODY 2).

Region	Nucleotide and systematic name	Protein effect	Reference
Islet promoter	c.−71G>C	—	[25]
Exon 2	c.106C>T	p.Arg36Trp	[26]
	c.130G>A	p.Gly44Ser	[27]
	c.157G>T	p.Ala53Ser	[20]
	c.182A>C	p.Tyr61Ser	[28]
	c.185T>C	p.Val62Ala	[21]
	c.184G>A	p.Val62Met	[21, 29]
	c.208G>A	p.Glu70Lys	[20, 30]
Exon 3	c.214G>A	p.Gly72Arg	[29]
	c.239G>C	p.Gly80Ala	[20]
	c.323A>G	p.Tyr108Cys	[21]
Exon 4	c.391T>C	p.Ser131Pro	[31]
	c.410A>G	p.His137Arg	[20]
	c.437T>G	p.Leu146Arg	[21]
	c.480_482dupTAA	p.Asp160_Lys161 ins Asn	[32]
Exon 5	c.493C>T	p.Leu165Phe	[33]
	c.502A>C	p.Thr168Pro	[20]
	c.523G>A	p.Gly175Arg	[20]
	c.524G>A	p.Gly175Glu	[20]
	c.544G>T	p.Val182Leu	[28]
	c.562G>A	p.Ala188Thr	[31]
	c.563C>A	p.Ala188Glu	[21]
Exon 6	c.608T>C	p.Val203Ala	[30]
	c.617C>T	p.Thr206Met	[33]
	c.622G>A	p.Ala208Thr	[22]
	c.626C>T	p.Thr209Met	[26]
	c.629T>C	p.Met210Thr	[20]
	c.637T>C	p.Cys213Arg	[20]
	c.676G>A	p.Val226Met	[20]
	c.682A>G	p.Thr228Ala	[34]
	c.697T>C	p.Cys233Arg	[28]
	c.703A>G	p.Met235Val	[32]
	c.704T>C	p.Met235Thr	[21]
	c.755G>A	p.Cys252Tyr	[21]
	c.766G>A	p.Glu256Lys	[35]
	c.769T>C	p.Trp257Arg	[31]
	c.781G>A	p.Gly261Arg	[20]
	c.787T>C	p.Ser263Pro	[21]
	c.793G>A	p.Glu265Lys	[28]
	c.823C>T	p.Arg275Cys	[21]
	c.835G>C	p.Glu279Gln	[35]
Exon 8	c.893T>A	p.Met298Lys	[21]
	c.895G>C	p.Gly299Arg	[35]
	c.898G>C	p.Glu300Gln	[35]
	c.898G>A	p.Glu300Lys	[30]
	c.922A>T	p.Arg308Trp	[32]
	c.926T>C	p.Leu309Pro	[20, 35]
	c.1007C>T	p.Ser336Leu	[20, 34]
	c.1016A>G	p.Glu339Gly	[21, 22]
Exon 9	c.1099G>A	p.Val367Met	[20, 26]
	c.1129C>T	p.Arg377Cys	[21, 22]
	c.1136C>T	p.Ala379Val	[28]
	c.1148C>T	p.Ser383Leu	[21]
	c.1232C>T	p.Ser411Phe	[21]
	c.1240A>G	p.Lys414Glu	[20, 36]
Exon 10	c.1258A>G	p.Lys420Glu	[28]
	c.1358C>T	p.Ser453Leu	[22]
	c.1364T>A	p.Val455Glu	[37]
